# Developing market class specific InDel markers from next generation sequence data in *Phaseolus vulgaris* L.

**DOI:** 10.3389/fpls.2014.00185

**Published:** 2014-05-13

**Authors:** Samira Mafi Moghaddam, Qijian Song, Sujan Mamidi, Jeremy Schmutz, Rian Lee, Perry Cregan, Juan M. Osorno, Phillip E. McClean

**Affiliations:** ^1^Genomics and Bioinformatics Program, North Dakota State UniversityFargo, ND, USA; ^2^Department of Plant Sciences, North Dakota State UniversityFargo, ND, USA; ^3^Soybean Genomics and Improvement Laboratory, United States Department of Agriculture, Agricultural Research ServiceBeltsville, MD, USA; ^4^HudsonAlpha InstituteHuntsville, AL, USA

**Keywords:** InDel marker, next generation sequencing, market class, phylogenetics, genetic map, common bean

## Abstract

Next generation sequence data provides valuable information and tools for genetic and genomic research and offers new insights useful for marker development. This data is useful for the design of accurate and user-friendly molecular tools. Common bean (*Phaseolus vulgaris* L.) is a diverse crop in which separate domestication events happened in each gene pool followed by race and market class diversification that has resulted in different morphological characteristics in each commercial market class. This has led to essentially independent breeding programs within each market class which in turn has resulted in limited within market class sequence variation. Sequence data from selected genotypes of five bean market classes (pinto, black, navy, and light and dark red kidney) were used to develop InDel-based markers specific to each market class. Design of the InDel markers was conducted through a combination of assembly, alignment and primer design software using 1.6× to 5.1× coverage of Illumina GAII sequence data for each of the selected genotypes. The procedure we developed for primer design is fast, accurate, less error prone, and higher throughput than when they are designed manually. All InDel markers are easy to run and score with no need for PCR optimization. A total of 2687 InDel markers distributed across the genome were developed. To highlight their usefulness, they were employed to construct a phylogenetic tree and a genetic map, showing that InDel markers are reliable, simple, and accurate.

## Introduction

Plant breeding embraces both art and science for crop improvement. Marker assisted selection (MAS) can boost the efficiency of breeding when markers linked to genes of interest are discovered (Yang et al., 2012). Marker development requires the comparison of genetic material of two or more genotypes to find the polymorphic regions that segregate in a breeding population. Thus, it is important to have adequate genetic variation among the genotypes of interest to develop markers that can be used for MAS and other genetic studies. In fact, using MAS to improve a trait of interest in a self-pollinating species like common bean is becoming more challenging today in the United States because of the narrow genetic diversity of this species (McClean et al., 1993; Sonnante et al., 1994).

Common bean is a diploid legume species with 11 chromosomes, a genome size of approximately 520 megabase pairs (Mbp), a few duplicated loci (Vallejos et al., 1992; Freyre et al., 1998) and 49% transposable elements (Schlueter et al., 2008). A reference genome sequence of genotype G19833 was recently released in August (Schmutz et al., in press). Common bean has two distinct gene pools, Middle American (from northern Mexico to Colombia) and Andean (from southern Peru to northwestern Argentina). Each gene pool underwent separate domestication events (Gepts and Bliss, 1986; Koenig and Gepts, 1989; Khairallah et al., 1990, 1992; Koinange and Gepts, 1992; Freyre et al., 1996) followed by the creation of ecogeographic races in each of the two gene pools due to further selection under domestication (Singh et al., 1991; Beebe et al., 2000; Diaz and Blair, 2006; Mamidi et al., 2011). Mamidi et al. (2011) estimated the duration and time of the single domestication event in each gene pool and suggested reciprocal migration between wild and landrace genotypes. There is a strong genetic differentiation between Middle American and Andean gene pools, and the Middle American gene pool is more diverse compared to the Andean gene pool (Mamidi et al., 2013). According to Singh et al. (1991), the Middle American gene pool with the center of domestication in Central and North America consist of three races, Durango, Jalisco, and Mesoamerican. The typical commercial market classes in the United States from this gene pool are pinto, great northern (GN), small red and pink beans from race Durango, and navy, small white and black beans from race Mesoamerican. The Andean gene pool with its center of domestication in South America includes three races: Nueva Granada, Peru, and Chile. The commercial market classes of this gene pool in the United States are light red kidney (LRK), dark red kidney (DRK), white kidney, and cranberry beans which are all from the Nueva Granada race (Mensack et al., 2010).

Breeding for commercial varieties in common bean usually occurs within each market class in order to retain their preferred seed size, shape, color, and pattern. The narrow genetic diversity within a market class is due to the small size of bottleneck populations (Gepts and Bliss, 1986), the rigid quality required by processors and consumers (Ghaderi et al., 1984; Wang et al., 1988; Hosfield et al., 2000; Myers, 2000), the finite use of exotic germplasm (Silbernagel and Hannan, 1988, 1992; Miklas, 2000), and the restricted breeding strategies to meet consumer satisfaction regarding seed size, shape, and color (Singh, 1992). Although incorporation of exotic and unadapted germplasm is helpful in enhancement of genetic diversity, maintenance of the necessary phenotypic characteristics of each market class is challenging when using novel sources of variation due to linkage drag. Indeed, it has been documented in multiple plant species that quantitative traits are affected by genetic background (Tanksley and Hewitt, 1988; Doebley et al., 1995; Lark et al., 1995; Cockerham and Zeng, 1996; Li et al., 1997, 1998). This indicates the need for market class specific marker development to facilitate bean improvement by monitoring the variation that exists in each market class.

Most of the currently available markers for common bean are Sequence Characterized Amplified Region (SCAR) markers developed from Random Amplification of Polymorphic DNA (RAPD) markers through a slow and difficult process (Kelly et al., 2003). Other types of marker systems have been developed and used in different studies in common bean such as Inter Simple Sequence Repeats (ISSR) (Gonzalez et al., 2005), Simple Sequence Repeats (SSR) (Blair et al., 2003; Gomez et al., 2004; Buso et al., 2006; Galeano et al., 2009; Cordoba et al., 2010). Recently a high-throughput Golden Gate SNP assay was released by Hyten et al. (2010). However, most of the markers are based on polymorphism among a few genotypes from different market classes or even gene pools. Thus, the development and application of high throughput, user-friendly, market class-specific markers are indispensable.

Insertion-deletions (InDel) are one of the common sources of variation that are distributed widely throughout the genome. Mechanisms such as transposable elements, slippage in simple sequence replication, and unequal crossover can result in the formation of InDels (Britten et al., 2003). They can be converted to user-friendly markers that can be distinguished easily based on their size (Vali et al., 2008) with minimum laboratory equipment. Many genetic studies in plants and animals have successfully utilized InDels (Hayashi et al., 2006; Vali et al., 2008; Vasemagi et al., 2010; Ollitrault et al., 2012). InDels and SNPs are now the most widely used marker systems in Arabidopsis because they are abundant, PCR-based, and informative due to their co-dominant nature (Pacurar et al., 2012).

Next generation sequencing (NGS) provides inexpensive sequence data needed to develop genetic markers to be used in plant breeding and genetic studies. NGS technologies are efficient and offer new genomic information for minor crops for which a reference genome sequence is not available (Varshney et al., 2009) and accelerates the development of genomic resources for crops with a reference genome. The objective of this study was to use Illumina sequence data from multiple genotypes within five bean market classes, which were selected from both the Andean and the Middle American gene pools, to develop user-friendly InDel markers that have wide applications for MAS and genomic studies.

## Materials and methods

### Plant materials

Three diverse genotypes from pinto, navy, black, and LRK and two genotypes from DRK market classes where sequenced with the Illumina Genome Analyzer (GAII). Genotypes were chosen based on their divergence in a neighbor joining (NJ) tree that was created for 192 genotypes from nine different market classes using 1159 high quality SNP markers (Hyten et al., 2010). The sequenced genotypes in each market class were as follows: Stampede, Buckskin, and Sierra from the pinto market class; C20, Michelite, and Laker from the navy market class; Cornell 49242, T-39, and UI 906 from the black market class, California Early, Lark and, Kardinal from the LRK market class, and Red Hawk and Fiero from the DRK market class.

### Marker development

The first step of marker design was a within genotype *de novo* assembly of the Illumina GAII DNA sequence data into contigs using Velvet 1.0 (Zerbino and Birney, 2008) with the default settings. BLAST+ (Camacho et al., 2009) was used to discover potential InDels. Using three genotypes within a market class, InDels were discovered by aligning contigs from one genotype as the query against a database consisting of the contigs of the two other genotypes. In addition, a pairwise blastn alignment was performed among all pairs of genotypes within a market class. An e-value cutoff of 1E-50 and a maximum hit of one and two were used in BLAST to obtain the best hit for pair-wise and three-way alignments, respectively. InDels were discovered based on the size differences between the query and the database subject. Several filters were applied to potential InDels: A minimum InDel size of 8 bp was used to ensure an appropriate resolution using agarose gel electrophoresis; unique InDel fragments were assured by blasting InDel fragments against the *Phaseolus vulgaris* L. scaffold assembly ARRA-V0.9. ARRA-V0.9 was an intermediate scaffold assembly in the whole genome sequencing project. The e-value and maximum hit were set to 1E-50 and 20, respectively. Queries with multiple hits were excluded to decrease the probability of designing markers from repetitive regions. Contigs that contained more than four consecutive Ns were excluded because this could lead to false InDel discovery or false InDel size.

The contigs that contained InDels were aligned using Multalin (Corpet, 1988) to obtain the consensus region around the InDels for primer design. Primers were designed from the consensus sequence in BatchPrimer3 (You et al., 2008). The primer size parameters were set to 22, 26, and 32 bp as the minimum, optimum, and maximum size, respectively, and GC content was set to 35, 50, and 60% as the minimum, optimum, and maximum, respectively. Primer annealing temperature was set to a very narrow range of 67, 68, and 69°C as the minimum, optimum, and maximum temperature, respectively. Finally, the maximum Tm difference between forward and reverse primers was set to 2°C only. The PCR product length was approximatly10 times the InDel size to ensure the PCR products could be adequately separated on a 3% agarose gel for efficient scoring. The product size varied between InDel size (bp) × 10 and [InDel size (bp) × 10] + 10 (bp) for optimum and maximum values, respectively. The minimum product sizes were as listed in Table 1. These stringent criteria were intentional to avoid the need for PCR optimization for each primer set. All primer sets were optimized to amplify with a 55°C annealing temperature.

**Table 1:** **InDel size and the corresponding minimum product size that was used by BatchPrimer3 for primer design**.

**InDel size (bp)**	**Minimum product size (bp)**
8–9	70
10–11	80
12–14	90
15–17	100
18–22	110
23–26	120
27–29	130
30–36	150

### Nomenclature

Including information on marker position on the reference genome in the marker names, provides the user with valuable information on marker distribution. Thus, the markers were named in the format NDSU-IND-NN-XX.XXXX, where NDSU stands for North Dakota State University, IND for InDels and NN for the chromosome number. The Xs represent the physical position on the reference genome (G19833- V1.0) in megabase pairs up to four decimals. For example InDel marker NDSU_IND_07_02.6485 is located on chromosome Pv07 at position 2.6485 Mbp in common bean V1.0 reference genome.

### PCR amplification

The PCR protocol used to amplify all InDel markers was: 3 min at 95°C for one cycle, 20 s at 95°C, 30 s at 55°C, and 1 min at 72°C for 45 cycles, and 10 min at 72°C for one cycle. Each PCR reaction consisted of a final concentration of 1× PCR buffer including 0.15 mM MgCl_2_, 0.5 mM dNTP mix, 0.25 mM forward/reverse primers and 1 unit of Taq polymerase with a 20 μl final volume.

### Alignment of sequence data with the reference genome

The sequence data from 14 genotypes were mapped to the reference genome (V1.0) when the complete assembly became available. Burrows-Wheeler Aligner (BWA) (Li and Durbin, 2009) with default settings was used to map the reads with the reference genome. SAMtools (Li et al., 2009) was used to convert the BWA output to a sorted bam file and obtain the mpileup file. The “pileup2indel” command with minimum coverage of five reads was used in VarScan (Koboldt et al., 2009) to find the number of InDels for each genotype based on the reference genome (G19833). The VarScan output was also filtered based on the frequency of the variant allele by read count. Only InDels with variant allele frequency of 80% and higher were considered.

### Marker performance and application

To evaluate the performance of the designed markers, 219 pinto markers were screened on Stampede, Sierra, Buckskin, and G19833. Moreover, six markers were tested on a few random genotypes from nine market classes (Table 2) to evaluate the performance of InDel markers in the market classes other than the one from which they were originally designed.

**Table 2:** **Genotypes used to test the performance of six markers in other market classes**.

Order^*^	**Genotype**	**Market class**
1	Domino	Black
2	Raven	Black
3	T-39	Black
4	Cornell 49242	Black
5	Shania	Black
6	Black Knight	Black
7	BelMiNeb-RMR-3	Great northern
8	Matterhorn	Great northern
9	Tara	Great northern
10	Coyne	Great northern
11	JM-24	Great northern
12	Gemini	Great northern
13	Michelite	Navy
14	Sanilac	Navy
15	Seafarer	Navy
16	Bunsi	Navy
17	C20	Navy
18	Laker	Navy
19	Pink Floyd	Pink
20	Victor	Pink
21	Viva	Pink
22	Roza	Pink
23	Gloria	Pink
24	PK915	Pink
25	AC Redbond	Small red
26	AC Earlired	Small red
27	Sapphire	Small red
28	Ember	Small red
29	UI-3	Small red
30	NW-63	Small red
31	Sierra	Pinto
32	Buckskin	Pinto
33	Durango	Pinto
34	PT7-2	Pinto
35	Lariat	Pinto
36	Hatton	Pinto
37	Chinook 2000	Light red kidney
38	K-42	Light red kidney
39	Blush	Light red kidney
40	VA-19	Light red kidney
41	Montcalm	Dark red kidney
42	USDK-CBB-15	Dark red kidney
43	Fiero	Dark red kidney
44	CDRK	Dark red kidney
45	Benton	Snap bean
46	91-G	Snap bean
47	Harvester	Snap bean
48	Cantar	Snap bean

Six random genotypes were selected from pinto, great northern, navy, black, pink, and small red market classes and four random genotypes were selected from dark and light red kidney and snap bean marker classes.

* Indicates the order of the genotypes from left to right on the agarose gel in Figure 3.

To assess the InDel markers for applied genetic studies, 196 markers were used to screen 24 diverse pinto genotypes. The 24 pinto genotypes were as follow: Sierra, Aztec, Santa Fe, La Paz, Stampede, ND-307, Medicine Hat, Lariat, BelDakMe-RR-5, Sequoia, Remington, Durango, Max, PT7-2, Ouray, JM-126, Olathe, Hatton, Apache, UI-114, Nodak, Buckskin, Flint, and UI-196. These genotypes were chosen based on their diversity in a NJ tree which was constructed in ClustalX 2.1 (Larkin et al., 2007) using 1300 SNP markers (Hyten et al., 2010). The number of bootstraps used in ClustalX was 1000 and the genotypes were chosen from clusters that were diverse and had high bootstrap values in the tree. The 24 pinto genotypes were screened using the 196 markers and the markers that showed polymorphism were used to evaluate the performance of the InDel markers for distinguishing the 24 genotype and to construct a NJ tree. PowerMarker version 3.25 (Liu and Muse, 2005) was used to construct the NJ tree with 1000 bootstraps. The *F*_st_ value was calculated in PowerMarker as well to evaluate the overall genetic divergence among this collection of genotypes.

In addition, an F_2_ population was used to evaluate the InDel markers for mapping purposes. The F_2_ population, NDZ-11002 was derived from a cross between Lariat × Medicine Hat and consisted of 87 F2 genotypes. Eighty two pinto markers that were polymorphic between the two parents were employed to screen the F_2_ population, and CarthaGène (De Givry et al., 2005) was used to build the genetic map. In CarthaGène, the “group” command was used with a distance and LD threshold of 20 cM and 3.00, respectively to group markers into linkage groups. The “Buildfw 2 2 {} 1” command was used to order the markers on each linkage group and to obtain the map with the highest likelihood value. Qgene (Nelson, 1997) was then used to visualize the map images.

### Multiplexing

Multiplexing of InDel markers was conducted using two and four markers in the same reaction mix. A total of one duplex and six fourplex sets were tested on 96 Middle American genotypes. The protocol for four markers in a 20 μl reaction mix was as follow: 1× PCR Buffer including 0.15 mM MgCl_2_, 0.8 mM dNTP, 0.125 mM of each forward and reverse primer [total of 0.5 mM (0.125 × 4) forward and reverse primers] and 2 units of Taq polymerase. The protocol for multiplexing two markers was the same as above with an exception that 0.25 mM of each forward and reverse primer [total of 0.5 mM (0.25 × 2)] was used. The PCR amplification cycle was the same as the cycle used to amply a single marker. The resulting amplification products were visualized on a 3% agarose gel.

## Results

### Illumina sequencing, *de novo* assembly and primer design

The DNA sequence data consisted of 19.6 billion bases in the form of 114 bp paired-end reads from 250 to 400 bp size selected fragments of 14 genotypes. The 114 bp paired-end reads did not include Illumina adaptor sequences. The sequencing coverage ranged from 1.6× to 5.1× with an average of 3.7×. Laker and Kardinal had the lowest and highest number of raw GAII reads, 3,711,450 and 11,748,671 reads, respectively. Cornell 49242 with 261,313 and Stampede with 752,015 contigs had the smallest and largest number of assembled contigs, respectively. Only contigs 120 bp or greater were used for the assembly statistics. The mean contig length across all 14 genotypes was 322 bp. The N50 varied from 222 to 651 bp, with an average N50 value of 394 bp. The GC content ranged from 32.5 to 40.9% with an average of 34.4%. The minimum GC value of 35% was used to design primers in BatchPrimer3. The Illumina reads and assembly information are summarized in Table 3.

**Table 3:** **Illumina paired-end reads information and contig information after the *de novo assembly***.

	**Illumina GAII reads**			**Assembly**			
**Genotype**	**Market class**	**Number of reads (in millions)**	Genome coverage (x)^*^	**Total length of assembled contigs (Mbp)^+^**	**Number of assembled contigs**	**N50 (bp)**	**GC%**
Cornell 49242	Black	7.20	3.2	66.97	261,313	300	40.9
T-39	Black	8.42	3.7	178.16	527,965	422	32.6
UI 906	Black	6.44	2.8	107.99	400,652	320	33.3
Red Hawk	DRK	7.20	3.2	128.33	427,260	360	35.0
Fiero	DRK	7.61	3.3	146.58	470,693	373	34.0
California Early	LRK	7.52	3.3	145.26	461,881	387	34.2
Lark	LRK	10.30	4.5	199.12	531,149	501	33.8
Kardinal	LRK	11.74	5.1	238.71	534,298	651	32.5
C20	Navy	7.84	3.4	100.52	355,778	338	37.6
Michelite	Navy	9.47	4.1	170.02	504,441	426	34.3
Laker	Navy	3.71	1.6	81.10	381,399	222	33.8
Stampede	Pinto	10.20	4.5	214.71	752,015	330	33.2
Sierra	Pinto	9.58	4.2	178.36	532,937	409	32.7
Buckskin	Pinto	10.45	4.6	190.93	529,875	477	33.7

* The genome coverage was calculated based on a 521 Mbp genome size and 114 bp paired-end Illumina reads.

+ Contigs 120 bp or greater were used for the assembly statistics.

Alignment of the reads with the V1.0 reference genome for 14 genotypes indicated a range of 2330 to 45,770 InDels of 1 bp or greater across the genome. The minimum and maximum number of InDels belonged to the Laker and Buckskin genotypes, respectively (Table 4).

**Table 4:** **Number and distribution of InDels in each genotype when aligned with G19833**.

**Genotype**	**Market class**	**Genome coverage (x)**	**Number of InDels in alignment with G19833**	**InDel frequency per Mbp**	**Number of InDels in 1× coverage**
Cornell 49242	Black	3.2	9226	17.7	2901
T-39	Black	3.7	33,662	64.6	9049
UI 906	Black	2.8	13,681	26.3	4817
Red Hawk	DRK	3.2	6899	13.2	2169
Fiero	DRK	3.3	8961	17.2	2667
California Early	LRK	3.3	7017	13.5	2114
Lark	LRK	4.5	17,056	32.7	3749
Kardinal	LRK	5.1	27,122	52.1	5226
C20	Navy	3.4	11,205	21.5	3238
Michelite	Navy	4.1	31,455	60.4	7525
Laker	Navy	1.6	2330	4.5	1421
Stampede	Pinto	4.5	37,902	72.8	8404
Sierra	Pinto	4.2	32,199	61.8	7612
Buckskin	Pinto	4.6	45,770	87.9	9907

The numbers are based on InDel size of 1 bp and greater, and 521 Mbp genome size. VarScan was used to discover the InDel polymorphisms.

The number of aligned contigs (BLAST output) within a market class varied between 296,852 and 859,350 in the three-way alignments. The aligned contigs were filtered based on the InDel size, and those with a tentative size of 8 bp or greater were retained. This significantly reduced the number of sequences to analyze in the next step. For example, the number of contigs in the DRK market class dropped from 296,852 to 1378, and the reduction was from 859,350 to 9634 for pinto market class. Filtering for uniqueness of hits in the common bean reference scaffolds reduced the range of contigs from 450 in DRK to 6010 in pinto, with an average value of 2114 contigs across all market classes. The total number of consensus sequences submitted to BatchPrimer3 across all alignments (three-way and all pair-wise alignments) varied from 11,406 in the pinto to 324 in the DRK market class when fragments containing four consecutive Ns in their sequence were removed (Table 5). The basic properties of 11,406 pinto contigs submitted to BatchPrimer3 are summarized in Table 6.

**Table 5:** **Filtering criteria for contigs used for primer design**.

	**Number of contigs with InDels in three-way alignment**			**Numbers across all alignments**	
**Market classes**	**Blast hits**	**InDel size ≥8**	**Unique scaffold hit/InDel ≥8**	**Contigs submitted to Batch primer3**	**Number of primer pairs**
Pinto	859,350	9634	6010	11,406	1343
Black	342,085	1955	708	1913	292
Navy	507,147	1515	650	1867	323
LRK	834,726	5524	2754	5456	669
DRK	296,852 (pair-wise)	1378	450	324	60

The numbers are provided only for three-way alignment in all market classes except the dark red kidney market class.

**Table 6:** **Pre-analysis of 11,406 pinto contigs submitted to BatchPrimer3**.

**Item**	**Mean**	**Std. deviation**	**Min**	**Max**	**Coe. of variation (%)**
Sequence length (bp)	390.49	174.45	104	2680	44.67
GC contents (%)	29.04	5.92	4.45	53.21	20.38

**Figure 1:**
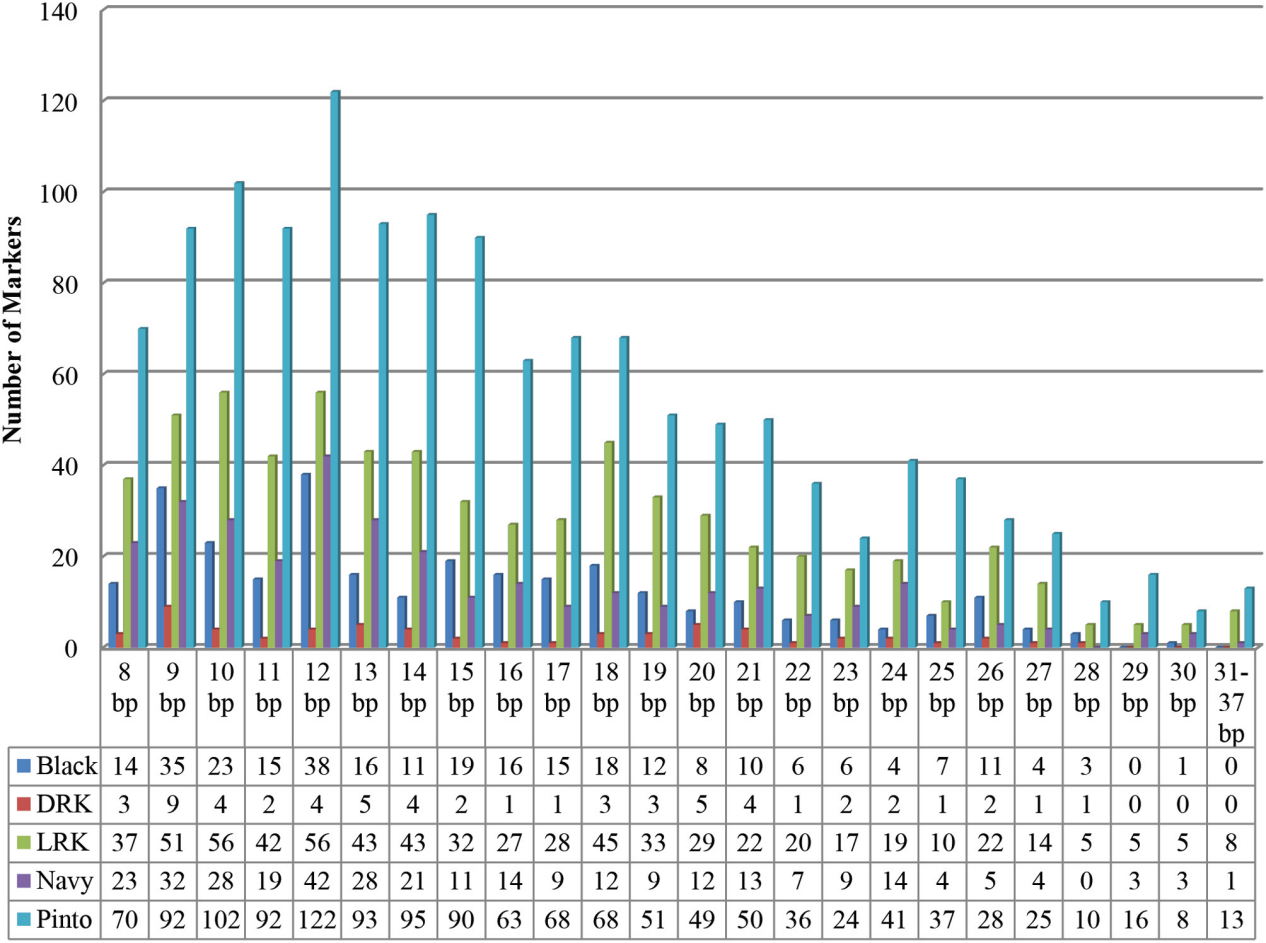
**Distribution of 2687 InDle sizes in five market classes**.

**Figure 2:**
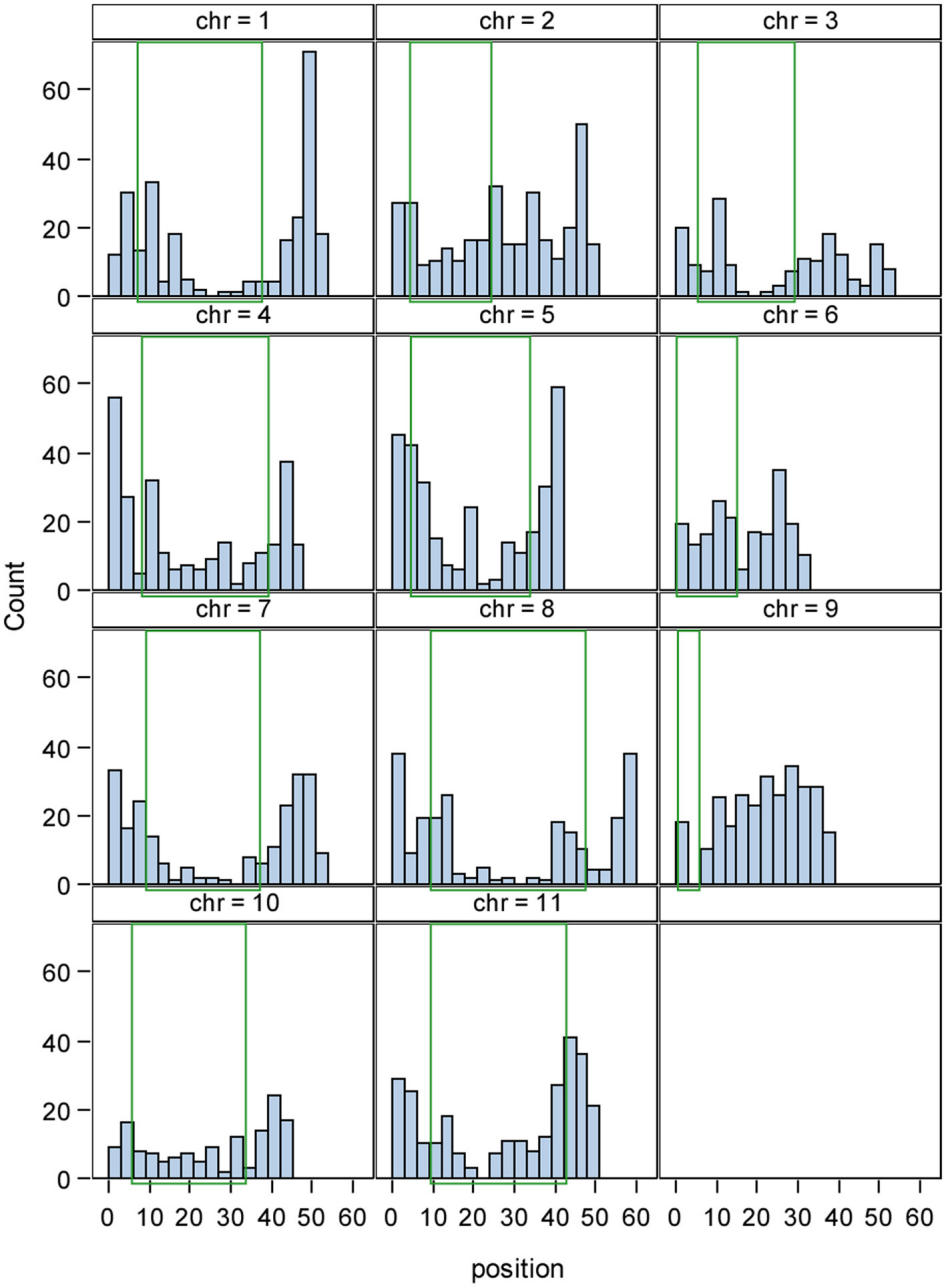
**Physical distribution of 2687 InDel markers across 11 chromosomes of common bean**. The x axis shows the chromosome length in Mbp and the y axis represents the frequency of InDel markers. The green rectangle indicates the pericentromeric region in each chromosome.

The final number of primer pairs ranged from 1343 in the pinto market class (highest) to 60 in the DRK market class (lowest) (Table 5). There were a total of 2687 primer pairs designed across all market classes (Supplementary Table), all having single hits on the common bean reference genome V1.0 and only six homologs to common bean transposable elements (Scott Jackson, personal communication). The distribution of InDel sizes in each market class is illustrated in Figure 1. The average distribution of InDel markers varied from one per132 Kb on chromosome Pv05 to one per 314 Kb on chromosome Pv03 with an average of one InDel marker every 200 Kb across the genome. Although the euchromatic region forms less than half of the bean genome (44.1%), the marker density was higher in this region (65.8%) (Figure 2). Markers located in the pericentromeric region are highlighted in orange color in the Supplementary Table. The number of markers on the chromosomes varied from 144 to 333 on chromosomes Pv10 and Pv02, respectively, with an average value of 238 markers per chromosome.

### Marker performance

**Figure 3:**
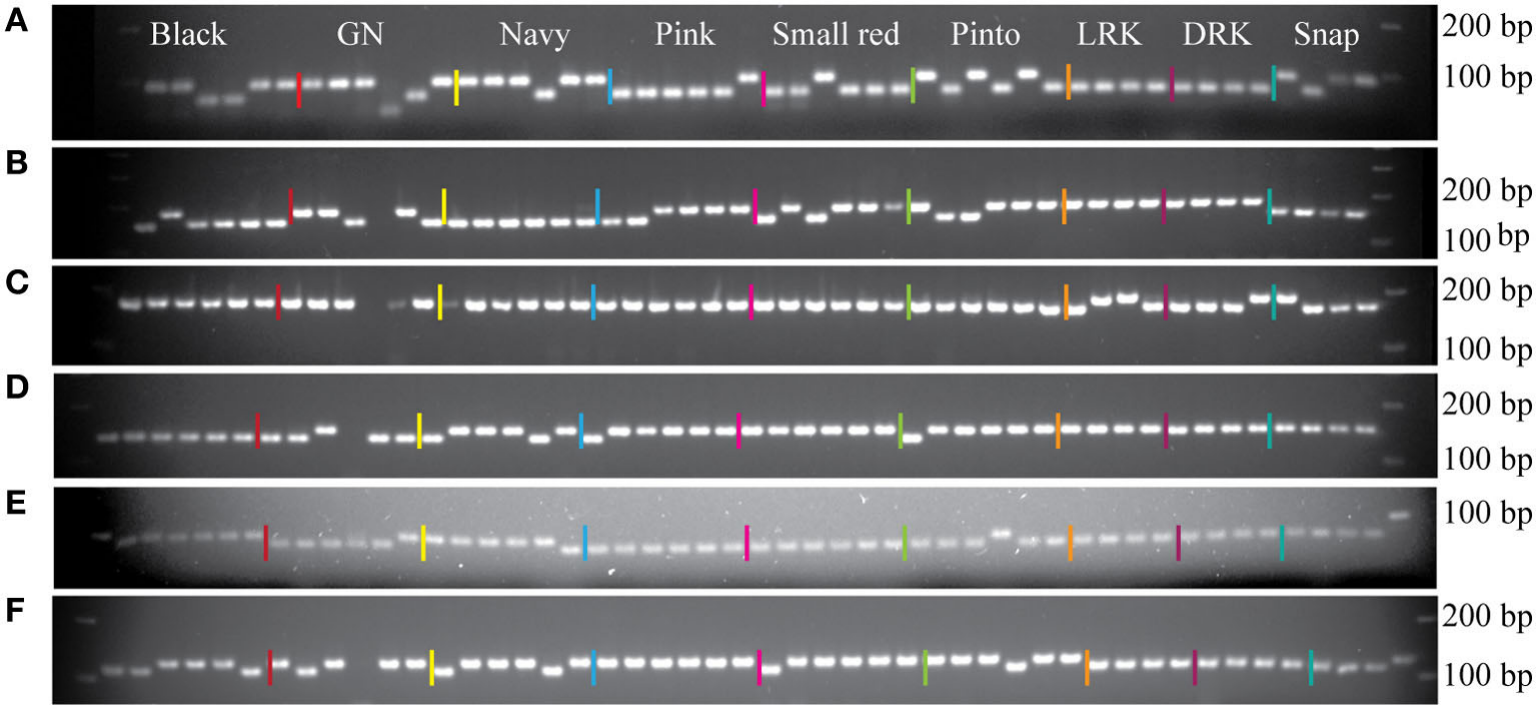
**Six InDel markers tested on random genotypes from nine different market classes**. The names of the genotypes, from left to right, are listed in Table 2. **(A)** Marker NDSU_IND_07_02.6485 from pinto market class. **(B)** Marker NDSU_IND_10_42.1355 from pinto market class. **(C)** Marker NDSU_IND_06_12.3324 from light red kidney market class. **(D)** Marker NDSU_IND_05_01.7405 from pinto market class. **(E)** Marker NDSU_IND_08_36.2119 from navy market class. **(F)** Marker NDSU_IND_09_07.6278 from black market class. First lane from right in all panels is the DNA Ladder.

To evaluate the performance of the InDel markers, a total of 219 markers from the pinto market class were tested with Stampede, Sierra, Buckskin, and G19833. A total of 196 markers showed polymorphism (89.5%), and only 23 markers (10.5%) were either monomorphic or difficult to score among four genotypes. Six markers from four market classes were tested on 48 random genotypes from nine different market classes as well. Although the primers were originally designed for a specific market class, we observed polymorphism among the genotypes from other market classes. Based on a sample of genotypes, markers from the Middle American gene pool did not show polymorphism in light and DRK market classes and the marker from the LRK market class showed polymorphism only among Andean genotypes (Figure 3).

### Marker application

#### Phylogenetic analysis

**Figure 4:**
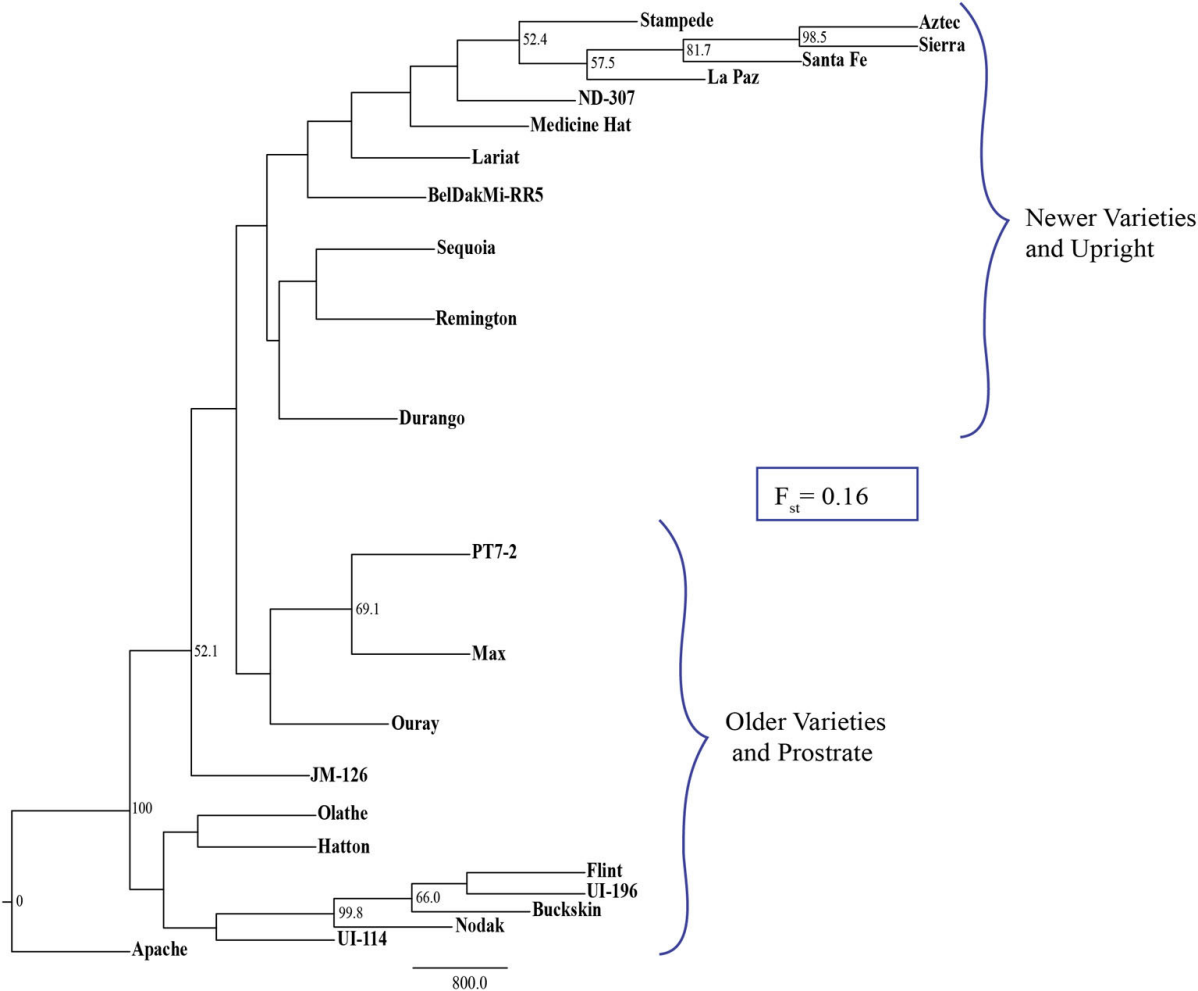
**Neighbor joining tree of 24 pinto genotypes that cluster into two distinct groups (i) newer varieties with type II growth habit and (ii) older varieties with type III growth habit**. The *F*_st_ value of 0.16 indicates the degree of variation between the two groups. Bootstrap values greater than 50% are shown on the nodes.

A set of 196 InDel markers were used to screen 24 diverse pinto genotypes (Table 7) and 172 (87.7%) were polymorphic and used in a phylogenetic analysis. The NJ tree and the *F*_st_ value indicated two distinct clusters among the 24 pinto genotypes (Figure 4).

**Table 7:** **Specifications of 24 pinto genotypes**.

**Genotype**	**Growth habit**	**Application date**	**Source**
PT7-2	II to III	Not released	USDA-ARS-Washington
Sequoia	IIb	NA	ISB
Max	III	NA	ISB
Santa Fe	IIa	2010	MSU
Stampede	IIa	2008	NDSU
La Paz	IIb	2008	ProVita, Inc.
ND-307	IIb	2008	NDSU
Lariat	IIb	2008	NDSU
Medicine Hat	IIa	2007	Seminis
Durango	IIb	2007	ProVita, Inc.
Remington	IIb	1996	RogersSeedCo
Hatton	IIIa	1996	NDSU
Buckskin	IIIb	1995	Novartis Seed Inc.
Apache	IIIa	1995	ISB
Aztec	IIb	1993	MSU
BelDakMi-RR-5	II	1993	USDA-ARS-Beltsville-MD
Sierra	IIb	1990	MSU
UI-196	IIIb	1990	UI
Flint	II to IIIb	1989	RogersSeedCo
JM-126	III to IIIa	1986	USDA-ARS/WSU
Nodak	III	1985	USDA-ARS/NDSU
Olathe	III	1980	CSU
Ouray	III to IIIa	1975	CSU
UI-114	III	1967	UI

NA, Information not available; ISB, Idaho Seed Bean Company; MSU, Michigan State University; NDSU, North Dakota State University; UI, University of Idaho; WSU, Washington State University; CSU, Colorado State University.

#### Genetic map

**Figure 5:**
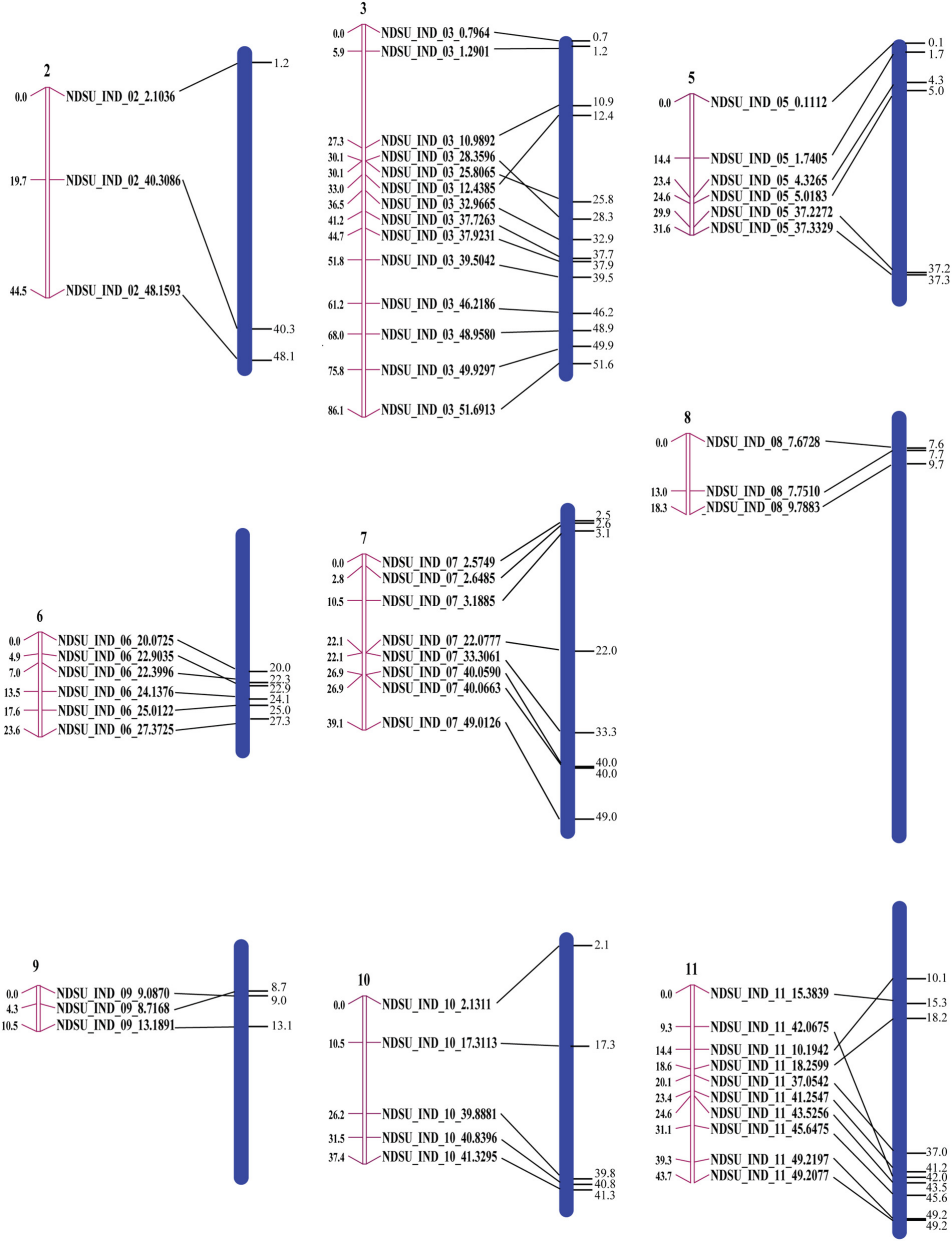
**Correspondence between genetic and physical positions**. The pink bars are linkage groups and the blue bars are the chromosomes with the physical positions of the InDel markers on their right side. The sizes of the chromosomes are proportional to their actual size.

Eighty two polymorphic InDel markers where used to construct a genetic map. A total of nine linkage groups that correspond to nine chromosomes (all chromosomes except one and four) were built from the F_2_ population with 87 genotypes. Five pairs of markers co-segregated in CarthaGène analysis. Among the 77 remaining markers, 18 were excluded from the genetic map when the “Buildfw” function of CarthaGène with a LOD threshold of 2.2 was used. The genetic and physical order was consistent for 54 of the 59 marker loci (Figure 5).

#### Multiplexing

**Figure 6:**
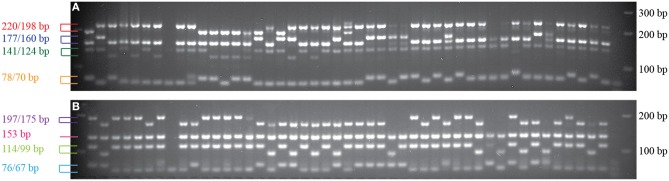
**Multiplexing of markers on 48 Middle American bean genotypes showed distinct bands on the 3% agarose gel electrophoresis. (A)** Amplification products using InDel markers NDSU_IND_05_37.2272, NDSU_IND_06_16.5002, NDSU_IND_11_30.9655, and NDSU_IND_06_31.8021 on 48 bean genotypes. All four markers showed polymorphism. **(B)** Amplification products using InDel markers NDSU_IND_11_33.0572, NDSU_IND_07_42.1709, NDSU_IND_07_25.1928, and NDSU_IND_10_19.1957 on the same 48 bean genotypes. Marker NDSU_IND_07_25.1928 was monomorphic and the other three were polymorphic. The first lane from the right in **(A,B)** are the DNA Ladders.

Multiplexing of tested InDel markers showed clear and scorable bands on the 3% agarose gel when one set of duplex and six sets of fourplex were used. Figure 6 illustrates the results of two fourplex sets on 48 Middle American genotypes on a 3% agarose gel as an example. The marker information is provided in Table 8.

**Table 8:**
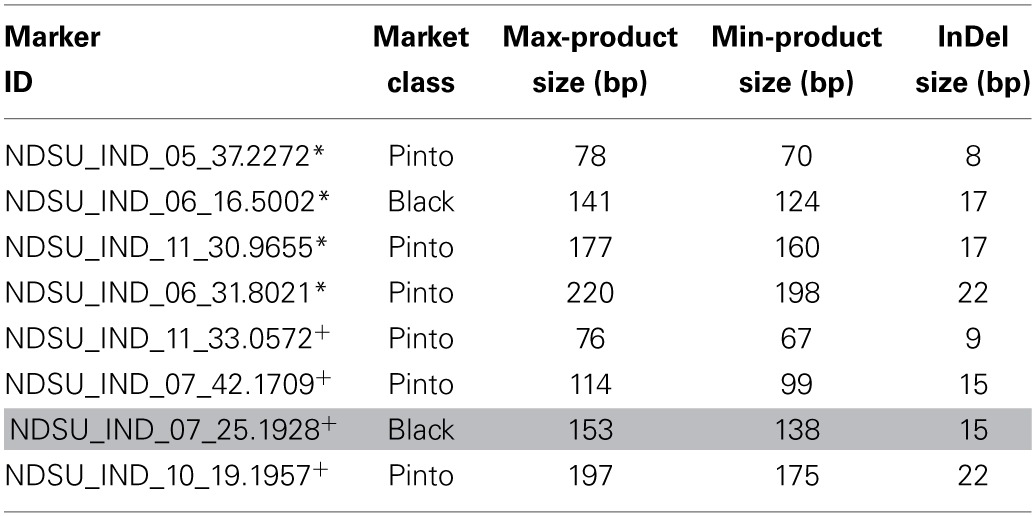
**Specifications of InDel markers used for multiplexing (two sets of fourplex)**.

Marker shaded in gray was monomorphic.

* One fourplex marker set mixed in one PCR reaction.

+ The second fourplex marker set mixed in one PCR reaction.

## Discussion

Common bean is a diverse crop species with much variation in the seed color, shape, and many other phenotypic characteristics. The species includes wild types, landraces which are the domesticated forms, ecogeographical races which are the result of selection, and market classes within each of the ecogeographical races. Plant breeding is generally restricted to market classes to retain the specific characteristics of the market class. However, as indicated by the high polymorphism rate (87.7%) based upon our analysis of 24 genotypes of the pinto market class, InDel markers appear to be polymorphic even within a market class. InDel markers are easy to use co-dominant markers and are present throughout the genome. With the availability of abundant next generation sequence data, identification of InDels has become a simple process.

### Marker development

We selected diverse genotypes in each market class based on the most comprehensive SNP dataset available. The Illumina GA II was used to generate paired end reads. The Illumina technology results in short reads but high coverage (Vera et al., 2008) as well as high quality data where 70% of base calls in 2 × 75 bp paired-end sequences have a quality score of Q30 or higher. The standard paired-end libraries of Illumina with a length between 200 and 500 bp can provide a platform to identify large and small InDels, inversions and other rearrangements. Paired-end reads boost the robustness of *de novo* assembly, SNP identification, and InDel discovery.

In this study we developed a genome wide collection of 2687 InDel markers that can be amplified without any PCR optimization and with minimum lab equipment. One of the filtering criteria that dramatically decreased the number of markers was the InDel size. There was a 215-fold decrease in the number of potential InDels in DRK when the contigs from the BLAST output were filtered for a minimum InDel size of 8 bp, and this reduction was about 89-fold for the pinto market class in the three-way alignment. However, filtering for uniqueness of hits to the common bean reference scaffolds (ARRA-V0.9) did not cause a dramatic reduction. For example, the number of contigs in the DRK market class dropped to one third, and this reduction was only 1.6-fold for the pinto market class in the three-way alignment. Generally there were less InDels in the DRK market class possibly due to the presence of only two sequenced genotypes in this market class. Moreover, Andean types are reported to have narrower genetic diversity compared to the Mesoamerican genotypes (Beebe et al., 2001). The stringent primer design criteria also resulted in another huge drop in the number of primer pairs that were selected. These stringent criteria were necessary because the development of markers should be precise and cost effective with proper throughput (Jander et al., 2002).

Several factors affect the discovery of functional InDel markers. As observed in Arabidopsis, decreasing InDel size from 25 to 6 bp increased the number of markers from 277 to 1073 (Salathia et al., 2007; Hou et al., 2010). The phylogenetic relationship between the genotypes used for InDel discovery is important. Kardinal, an Andean genotype, is more closely related to the reference genome (G19833), another Andean genotype, than Buckskin, a Middle American genotype. Less Kardinal InDels were observed than Buckskin InDels (27,122 vs. 45,770) even thought it had greater read coverage (5.1× vs. 4.6×). This trend was observed for all genotypes: more InDels were discovered among Middle American genotypes than the Andean genotypes because the reference genome is of Andean origin (Table 4).

In total we discovered 2687 InDel markers for an average of one per 200 Kb. The fact that they are preferentially distributed in the highly recombinogenic region of the genome increases their utility for multiple genetic analyses.

### Marker application

In our study, 87.7% of the 196 markers that were used in the phylogenetic analysis of 24 pinto genotypes were polymorphic. In the NJ tree, the pintos were separated based on plant architecture and the application/release date of the variety. Indeed, newer, upright pintos clearly clustered separately from older, prostrate pintos with fixation index (*F*_st_) of 0.16 which indicates a great degree of genetic divergence among subpopulations (Hartl and Clark, 1997). This might be due to selection for growth habit in bean breeding programs where the newer breeding programs prefer the upright beans since this trait offers several advantages such as ease of management, higher grain yield, and reduced disease issues (Cunha et al., 2005). InDel markers have been used for phylogenetic studies. Steele et al. (2008) used InDel polymorphisms in rice to separate Basmati genotypes from other genotypes. Ollitrault et al. (2012) showed that citrus diversity and phylogenetics based on InDel data are consistent with those based on SSR markers.

The observation that InDel markers developed from one market class showed polymorphism in the other market classes indicates their broad utility. This denotes that although these InDel markers were designed to capture the variation within each market class, their performance and application can be expanded to the entire bean germplasm.

The InDel markers should be useful for genetic map construction because there are on average about 200 markers on each chromosome. We used a relatively small mapping population to illustrate their application for linkage analysis. Generally, recombination occurs more frequently in regions distal to the centromeric region (Curtis and Lukaszewski, 1991; Tanksley et al., 1992; Werner et al., 1992). Because of low marker density and a small number of recombination events, our map did not cover the centromeric blocks in the F2 mapping study, resulting in mapping only a portion of the chromosome or of two clusters of markers, one from each arm. There were five discrepancies in our genetic map relative to the physical map. Marker order differences between the genetic and physical map or genetic maps from different populations or marker systems has been observed in other studies as well (Snelling et al., 2007; Wei et al., 2007; Xia et al., 2007). These differences could be a result of sequence assembly errors, inversions, and segregation distortion.

The possibility of conducting multiplex PCR is another indicator of the broad utility of InDel markers. With multiplexing, genotyping is even more cost effective due to reduced amount of reagents and DNA quantity needed for PCR amplification. Moreover, this method saves time when hundreds of markers are screened and broadens the coverage when DNA availability is limited (Edwards and Gibbs, 1994; Karaiskou and Primmer, 2008). Our InDel markers meet many of the criteria that Henegariu et al. (1997) mentioned as critical parameters in a multiplex PCR. According to their study, some of the basic principles include the appropriate primer length which should be 18–34 bp or higher and all of our primers are designed with a length of 26–32 bp. Henegariu et al. (1997) also reported that by increasing the primer length up to 28–30 bp, the annealing temperature could be increased resulting in a reduction of non-specific PCR products. GC content of 35–60% and annealing temperature between 55 and 58°C are other basic principles of multiplex PCR. The primers we designed have a minimum GC content of 35%, and all amplify at 55°C. Henegariu et al. (1997) indicated 54°C as the optimum temperature for co-amplification of loci in the multiplex PCR. Although the probability of non-specific product amplification increases at this temperature, simultaneous amplification of many specific loci greatly suppresses the yield of non-specific amplification products due to limited enzyme and nucleotide resources.

In conclusion, this study shows the usefulness of DNA sequence data as the raw material for primer development in the presence or absence of a reference genome. We show that contigs obtained from the *de novo* assembly of sequence data are sufficient for polymorphism discovery. However, without a completely assembled reference genome or a set of primary scaffolds, contigs cannot be filtered to eliminate the duplicate loci or transposable elements. The reference sequence could reduce the development of redundant markers and allow the determination of the exact physical position and order of the markers. The availability of high density markers affects the success of genetic map construction, map-based cloning (Pacurar et al., 2012) and diversity studies. The availability of 2687 InDel primers will enhance MAS and diversity studies in common bean.

### Conflict of interest statement

The authors declare that the research was conducted in the absence of any commercial or financial relationships that could be construed as a potential conflict of interest.
